# Use of fractional factorial design to study the compatibility of viral ribonucleoprotein gene segments of human H7N9 virus and circulating human influenza subtypes

**DOI:** 10.1111/irv.12269

**Published:** 2014-07-09

**Authors:** Alex W H Chin, Chris K P Mok, Huachen Zhu, Yi Guan, Joseph S M Peiris, Leo L M Poon

**Affiliations:** a Centre of Influenza Research and School of Public Health, The University of Hong KongHong Kong SAR, China; b HKU-Pasteur Pole, School of Public Health, The University of Hong KongHong Kong SAR, China

**Keywords:** Fractional factorial design, H7N9, influenza polymerase

## Abstract

Avian H7N9 influenza viruses may pose a further threat to humans by reassortment with human viruses, which could lead to generation of novel reassortants with enhanced polymerase activity. We previously established a novel statistical approach to study the polymerase activity of reassorted vRNPs (Influenza Other Respir Viruses. 2013;7:969-78). Here, we report the use of this method to study recombinant vRNPs with subunits derived from human H1N1, H3N2, and H7N9 viruses. Our results demonstrate that some reassortant vRNPs with subunits derived from the H7N9 and other human viruses can have much higher polymerase activities than the wild-type levels.

## Background

Coinfections and reassortments of influenza viruses play critical roles in the genesis of influenza pandemics. All pandemic strains from the last three pandemics have chimeric viral ribonucleoprotein (vRNP) complexes with subunits derived from different hosts.^1^,^2^ Extensive studies from the last two decades also indicated that vRNP complex can control viral virulence and interspecies transmission.^3^–^5^ Molecular markers associated with host adaptations in vRNP complex were also identified.^6^,^7^

The recent detection of avian H7N9 influenza virus in humans is of great concern. The virus was controlled in May 2013 after poultry market closures and other interventions. However, the detection of new human cases since October 2013 suggests that this virus is still circulating in poultry and that zoonotic transmission to humans may increase in winter season, when avian influenza activity in poultry is known to increase.^8^ Alarmingly, coinfections of avian H7N9 and human H3N2 viruses have been detected.^9^ It is possible that the H7N9 virus may generate a reassortant of pandemic potential by reassortment with seasonal human influenza viruses.

## The study

**Figure 1:**
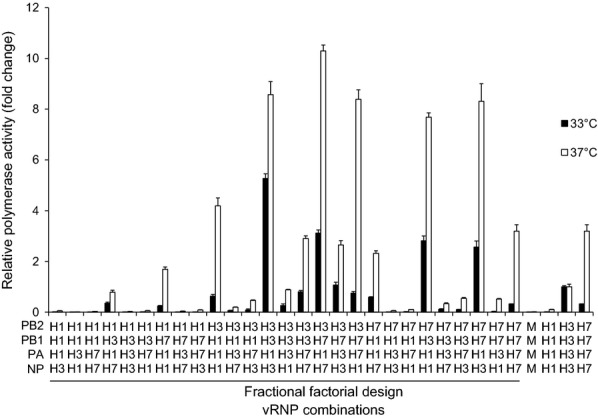
Mean relative polymerase activity of chimeric vRNPs of different viruses from the 27-run fractional factorial design at different temperatures. Twenty-seven chimeric vRNP combinations originated from human H1N1 (H1), human H3N2 (H3), and human isolate of avian H7N9 (H7) viruses were selected from the 27-run fractional factorial design. The chimeric vRNPs were reconstituted in 293T cells at 33°C (black) and 37°C (white). Mock transfection (M) and wild-type H1N1, H3N2, and H7N9 vRNPs were used as control. The polymerase activities of the chimeric vRNPs were studied by luciferase reporter assay. The normalized data are expressed as mean relative polymerase activity in relative to the polymerase activity of wild-type H3N2 vRNP at the corresponding temperatures (*n* = 3). Error bars represent one standard deviation.

We have recently reported the use of fractional factorial design and statistical methods to analyze the polymerase activity of recombinant vRNPs derived from three different strains. By only characterizing a subset of recombinant vRNPs (27 of all 81 possible combinations), we can use multiway anova to identify factors, including single vRNP subunits and interactions between two vRNP subunits, that control viral polymerase activity.^10^ Essentially, this statistical approach can identify subunit combinations that can yield robust polymerase activity without testing all possible vRNP combinations. Here, we aim at applying this strategy to a panel of reassorted vRNPs with subunits derived from the human H7N9 (A/Shanghai/2/2013) virus and human influenza viruses (H1N1: A/California/4/2009 and H3N2: A/HK/405595/2009). In brief, 27 different chimeric vRNP combinations were chosen by the 27-run fractional factorial design (Figure1) and were reconstituted in human 293T cells as described.^10^ The H1N1, H3N2, and H7N9 wild-type vRNPs were also reconstituted as controls. The transfected cells were incubated at 33°C and 37°C for 48 hours before determining their polymerase activity. All experiments were conducted in triplicate. Among the three wild-type vRNPs, the H3N2 vRNP had the highest polymerase activity at 33°C. For the easy identification of recombinant vRNPs with enhanced polymerase activity, all the polymerase activities were expressed in relative to the H3N2 vRNP activity at the corresponding temperature (Figure1; see Supplementary Figure for data without normalization). To identify vRNP subunits or two-factor interactions that are important for modulating polymerase activity, the deduced data were analyzed by multiway anova as described.^10^

Our statistical analysis indicated that the origin of the PB2 or PA gene segment is important for modulating polymerase activity. Both H7N9 and H3N2 PB2 could stimulate polymerase activity as compared to H1N1 PB2 at 33°C and 37°C (*t*-test, *P* < 0·01). Furthermore, H7N9 PA was found to have a moderate stimulatory effect as compared to H3N2 PA at 37°C (*t*-test, *P* < 0·05), while H1N1 PA was found to have the strongest stimulatory effect on polymerase activity at both temperatures (*t*-test, *P* < 0·001). The introduction of H7N9 PB1 or NP did not have a significant effect on polymerase activity at either temperature. We also validated the above findings by conducting an independent experiment using another fractional factorial design (i.e., another subset of 27 chimeric vRNPs with different gene segment combinations). The conclusions drawn from this independent vRNP panel are identical to those as reported above (data not shown). Overall, these results suggest that the vRNP gene segments of H7N9 virus are generally well adapted in human cells and can readily reassort with vRNP gene segments from human H1N1 and H3N2 viruses.

**Figure 2:**
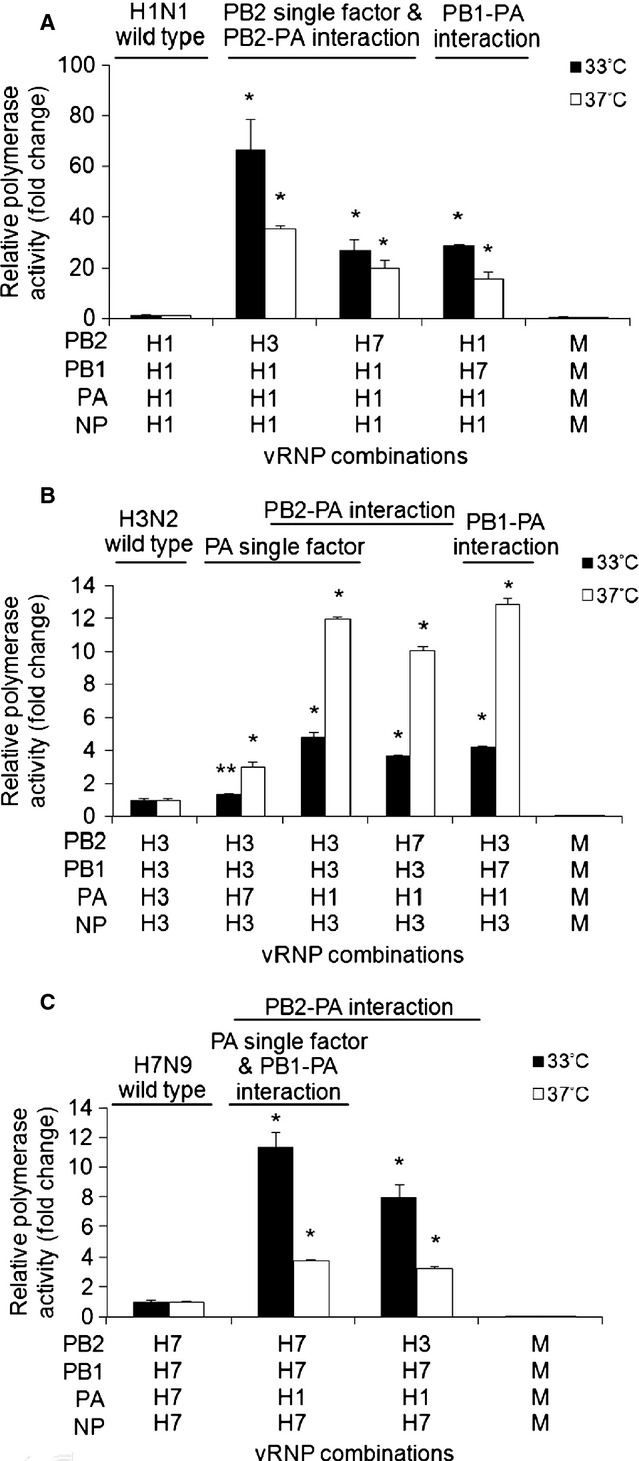
Mean relative polymerase activity of wild-type and chimeric vRNPs at different temperatures. Wild-type (A) H1N1 (H1), (B) H3N2 (H3), or (C) H7N9 (H7) and chimeric vRNPs with different subunits were reconstituted in 293T cells at 33°C (black) and 37°C (white). Mock-treated cells (M) were used as controls. The polymerase activities of the chimeric vRNPs were determined by luciferase reporter assay. The normalized data are expressed as mean relative polymerase activity in relative to the polymerase activity of wild-type (A) H1N1, (B) H3N2 or (C) H7N9 vRNP at the corresponding temperatures, respectively (*n* = 3). Error bars represent one standard deviation. (*P < 0·01, **P < 0·05, by *t*-test).

To further confirm our observations, we generated a series of chimeric vRNPs that are predicted to have robust polymerase activities (Figure2). When the PB2 of H1N1 vRNP was replaced by H3N2 or H7N9 PB2, the polymerase activity increased significantly at both 33°C and 37°C as predicted (Figure2A, PB2 single factor). Replacing the PA of H3N2 vRNP by H1N1 or H7N9 PA revealed that both H1N1 and H7N9 PA could enhance polymerase activity of the H3N2 vRNP at both temperatures (Figure2B, PA single factor). In agreement with our statistical analysis, the stimulatory effect caused by H1N1 PA in the H3N2 vRNP was found to be more pronounced than that induced by the PA of H7N9 (Figure2B, PA single factor). This was further confirmed by the introduction of H1N1 PA into the H7N9 vRNP, which showed that H1N1 PA can enhance polymerase activity (Figure2C, PA single factor).

Two-factor interactions which lead to enhanced polymerase activity were also identified in our statistical analysis. Our analysis revealed that the PB2–PA combination had the most significant effect on determining the polymerase activity. The analysis indicated that recombinant vRNPs with H7N9/H3N2 PB2 and H1N1 PA have robust polymerase activities (*t*-test, *P* < 0·05). In addition, the PB1–PA combination at 37°C was also found to be statistically significant (*t*-test, *P* < 0·05), and vRNPs containing H7N9 PB1 and H1N1 PA were predicted to have robust polymerase activity. To confirm these findings, we studied chimeric vRNPs with these gene combinations (Figure2). It was observed that the introduction of H3N2/H7N9 PB2 and H1N1 PA into the wild-type vRNPs could significantly enhance polymerase activity (Figure2A–C, PB2–PA interaction). Furthermore, when H7N9 PB1 and H1N1 PA were introduced into wild-type vRNPs, the polymerase activity of the reassorted vRNPs was also enhanced (Figure2A–C, PB1–PA interaction). Overall, these data agree with our predictions.

**Figure 3:**
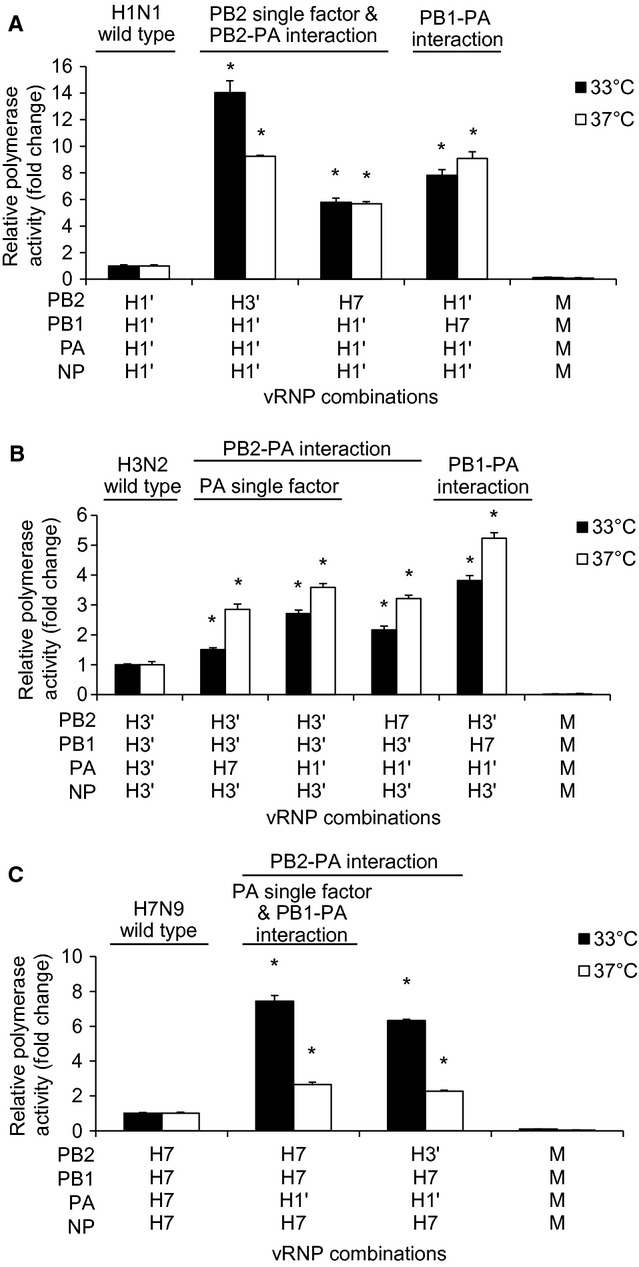
Mean relative polymerase activity of wild-type and chimeric vRNPs at different temperatures. Wild-type (A) H1N1 (A/HK/4259592/2013; H1′), (B) H3N2 (A/HK/4003076/2013; H3′), or (C) H7N9 (H7) and chimeric vRNPs with different subunits were reconstituted in 293T cells at 33°C (black) and 37°C (white). Mock-treated cells (M) were used as controls. The polymerase activities of the chimeric vRNPs were determined by luciferase reporter assay. The normalized data are expressed as mean relative polymerase activity in relative to the polymerase activity of wild-type (A) H1N1, (B) H3N2, or (C) H7N9 vRNP at the corresponding temperatures, respectively (*n* = 3). Error bars represent one standard deviation (*P < 0·01 by *t*-test).

To test whether the above-identified factor or combinations would have similar stimulatory effects on chimeric vRNPs with subunits derived from H7N9 and recently circulating human viruses, the vRNP gene segments of A/HK/4259592/2013 (H1N1) and A/HK/4003076/2013 (H3N2) were cloned and further studied. Recombinant vRNPs with gene segment combinations identical to those as described in Figure2 were generated and characterized. Data from this new set of chimeric vRNPs generally agreed with the predictions. All the factors or combinations predicted to have stimulatory effects in our initial analyses were found to stimulate polymerase activity (Figure3). It is worth noting that there are some very minor differences between these two set of chimeric vRNPs in terms of the degree of enhancement. The vRNPs of A/HK/4259592/2013 and A/California/4/2009 have a total of 18 amino acid differences, whereas the vRNPs of A/HK/4003076/2013 and A/HK/405595/2009 have 16 differences (Supplementary Table). Further mutagenic study on these positions might help to explain the effect of these subtle variations on the polymerase activities. Nonetheless, these results demonstrated that our predictions are still applicable to contemporary seasonal viruses.

The H7N9 virus emerged and reemerged in humans in the last several months. The chance of coinfection of seasonal and H7N9 viruses in humans (or other mammalian species) and the potential for emergence of novel reassortants of H7N9 virus should not be overlooked. In fact, coinfections and reassortments of influenza viruses have been detected in humans in the past.^11^–^13^ The detection of a coinfection of avian H7N9 and seasonal H3N2 viruses in human is of huge public health concern.^9^ It should also be noted that the hemagglutinin of H7N9 readily binds to alpha-2,6 sialic acid receptor, which typically indicates the potential for human–human transmission.^14^ It is possible that H7N9 might acquire polymerase gene segment(s) from human influenza viruses, resulting in a novel reassortant which is highly adapted in humans.

Viral polymerase complexes with H3N2/H7N9 PB2 and H1N1 PA were shown to have enhanced polymerase activity at both 33°C and 37°C, suggesting that viruses with these gene segment combinations might well adapted in both human upper and lower respiratory tracts. Sequence analysis of genes used in this study reveals that the H3N2 and H7N9 PB2 gene segments both have 627K, which is responsible for high polymerase activity in human cells, while the H1N1 PB2 possesses the avian marker 627E. The PB2-627 polymorphism may partly explain the effect of PB2 on polymerase activity observed in this study. On the other hand, the PA of pandemic H1N1/2009 possesses three signature mutations (T85I, G186S, and L336M) that might enhance polymerase activity.^15^ However, it is not sure whether these PB2 and PA mutations have a synergistic effect in enhancing polymerase activity. The primary objective of this work was to study the compatibility of vRNP subunits derived from the representative strains selected, and further systematic mutagenic studies will be needed to reveal how the interactions between different subunits can affect polymerase activity.

To monitor the emergence of any new reassortant that may lead to a new wave of epidemic or even a pandemic in humans, all vRNP gene segments from human and animal H7N9 cases should be at least partially sequenced. In particular, our results showed that the introduction of PA of pandemic H1N1 lineage can dramatically stimulate the polymerase activity. Attention should therefore be paid to future H7N9 reassortants which carry a PA derived from the pandemic H1N1/2009 lineage.

## Supporting Information

**Figure S1.** Mean polymerase activity of chimeric vRNPs of different viruses from the 27-run fractional factorial design at different temperatures. **Table S1.** Amino acid differences between seasonal and prototype vRNPs used in this study.
