# Supplementary material for: Use of fractional factorial design to study the compatibility of viral ribonucleoprotein gene segments of human H7N9 virus and circulating human influenza subtypes
Source: Influenza Other Respir Viruses. 2014 Jul 9;8(5):580–4. doi: 10.1111/irv.12269 (PMC4161617; doi:10.1111/irv.12269)
Supplement: Supplementary file 1 [file irv0008-0580-SD1.pptx]

## Slide 1
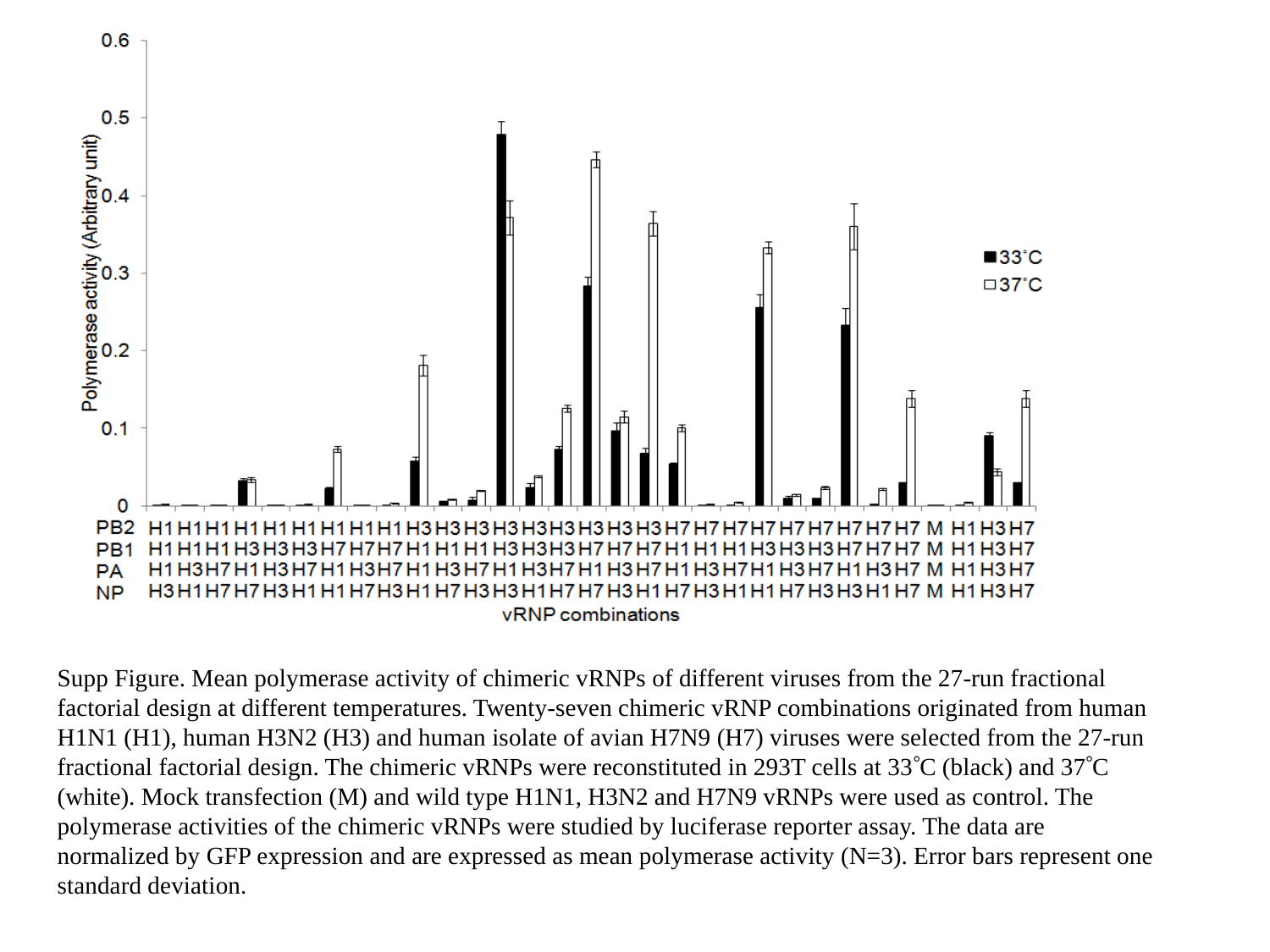

Supp Figure. Mean polymerase activity of chimeric vRNPs of different viruses from the 27-run fractional factorial design at different temperatures. Twenty-seven chimeric vRNP combinations originated from human H1N1 (H1), human H3N2 (H3) and human isolate of avian H7N9 (H7) viruses were selected from the 27-run fractional factorial design. The chimeric vRNPs were reconstituted in 293T cells at 33°C (black) and 37°C (white). Mock transfection (M) and wild type H1N1, H3N2 and H7N9 vRNPs were used as control. The polymerase activities of the chimeric vRNPs were studied by luciferase reporter assay. The data are normalized by GFP expression and are expressed as mean polymerase activity (N=3). Error bars represent one standard deviation.

## Slide 2
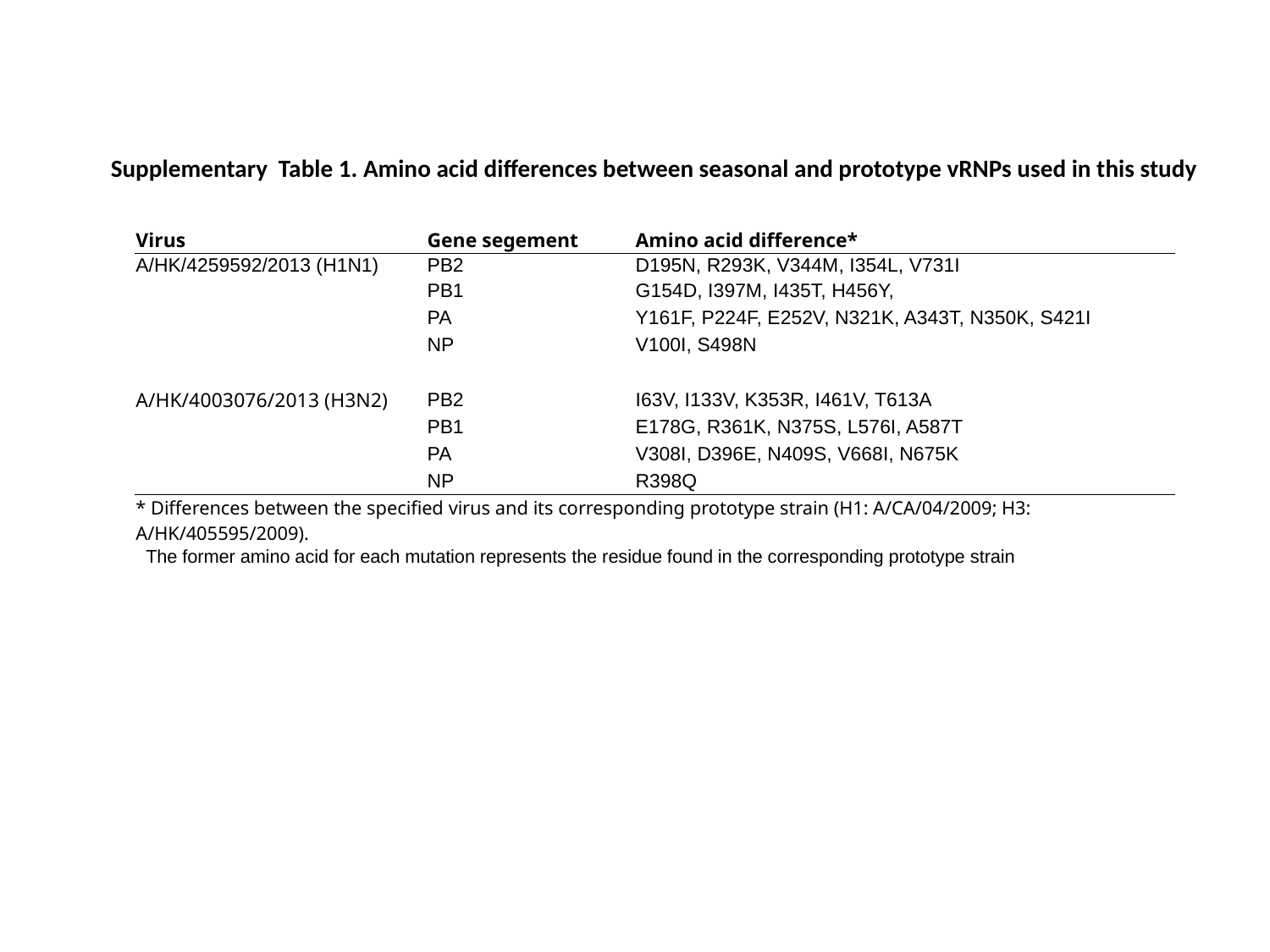

Supplementary Table 1. Amino acid differences between seasonal and prototype vRNPs used in this study
| Virus | Gene segement | Amino acid difference\* |
| --- | --- | --- |
| A/HK/4259592/2013 (H1N1) | PB2 | D195N, R293K, V344M, I354L, V731I |
| | PB1 | G154D, I397M, I435T, H456Y, |
| | PA | Y161F, P224F, E252V, N321K, A343T, N350K, S421I |
| | NP | V100I, S498N |
| | | |
| A/HK/4003076/2013 (H3N2) | PB2 | I63V, I133V, K353R, I461V, T613A |
| | PB1 | E178G, R361K, N375S, L576I, A587T |
| | PA | V308I, D396E, N409S, V668I, N675K |
| | NP | R398Q |
| \* Differences between the specified virus and its corresponding prototype strain (H1: A/CA/04/2009; H3: A/HK/405595/2009). | | |
| The former amino acid for each mutation represents the residue found in the corresponding prototype strain | | |
